# Diabetes Mellitus and Chronic Kidney Disease: The Future Is Being Surpassed

**DOI:** 10.3390/jcm14238326

**Published:** 2025-11-23

**Authors:** Alberto Martínez-Castelao, José Luis Górriz, Beatriz Fernández-Fernández, María José Soler, Juan F. Navarro-González

**Affiliations:** 1 Nephrology Department, Bellvitge University Hospital, Hospitalet, 08907 Barcelona, Spain; 2 GEENDIAB (Grupo Español de Estudio de la Nefropatía Diabética), Sociedad Española de Nefrología (S.E.N.), 39008 Santander, Spain; bfernandez@fjd.es; 3 RED in REN-RICORS 2040, Instituto de Salud Carlos III, 28029 Madrid, Spain; 4 Servicio de Nefrología, INCLIVA, Universidad de Valencia, 46010 Valencia, Spain; jlgorriz@gmail.com; 5 GEENDIAB-S.E.N., 39008 Santander, Spain; mjsoler01@gmail.com (M.J.S.); jnavgon@gobiernodecanarias.org (J.F.N.-G.); 6 RICORS2040 (RD24/004/0022), Instituto de Salud Carlos III, 28029 Madrid, Spain; 7 Servicio de Nefrología, Fundación Jiménez Díaz, 28040 Madrid, Spain; 8 RICORS2040-Renal, Instituto de Salud Carlos III, 28029 Madrid, Spain; 9 Servicio de Nefrología, Hospital Universitari Vall d’Hebrón, 08035 Barcelona, Spain; 10 Unidad de Investigación y Servicio de Nefrología, Hospital Universitario Nuestra Señora de Candelaria, 38010 Santa Cruz de Tenerife, Spain; 11 Instituto de Tecnologías Biomédicas, Universidad de La Laguna, 38010 Santa Cruz de Tenerife, Spain; 12 Facultad de Ciencias de la Salud, Universidad Fernando Pessoa Canarias, 35450 Las Palmas de Gran Canaria, Spain

**Keywords:** diabetes mellitus, diabetic kidney disease, chronic kidney disease, obesity, new hypoglycemic agents

## Abstract

Diabetes mellitus (DM) continues to be a global world health problem. Despite medical advances, both DM and chronic kidney disease (CKD) remain global health issues with high mortality and limited options to prevent end-stage renal failure. Current therapies encompass five classes of drugs: (1) angiotensin-converting-enzyme inhibitors (ACEI) or angiotensin II receptor blockers (AIIRB); (2) sodium-glucose-transporter 2 (SGLT2) inhibitors; (3) glucagon-like peptide-1 receptor agonists (GLP-1 RA); and (4) an antagonist of type 1 endothelin receptor (ET1R) with proven efficacy to reduce albuminuria and proteinuria. (5) The mineralocorticoid receptor antagonist (MRA) finerenone has been tested in RCTs as a kidney protective agent. In our review, we summarize many of the principal trials that have generated evidence in this regard. Many novel agents—many of them proven not only for DM management but also for the treatment of obesity with or without DM or heart failure (HF)—are now in development and may be added to the five classical pillars: other non-steroidal MRA (balcinrenone); aldosterone synthase inhibitors (baxdrostat and vicadrostat); other GLP-1 RA (tirzepatide, survodutide, retatrutide, and cagrilintide); ET1 R antagonists, (zibotentan); and soluble guanylate cyclase activators (avenciguat). These new agents aim to slow disease progression further and reduce cardiovascular risk. Future strategies rely on integrated, patient-centered approaches and personalized therapy to curb renal disease and its related complications.

## 1. Introduction

Diabetes mellitus (DM) continues to be a global world health problem. In November 2024, the global disease burden of DM prevalence and treatment from 1990 to 2022 was published in *The Lancet*. A pooled analysis of 1108 population-representative studies with 141 million participants aged above 18 y.o. estimated that there have been 630 million (554–713) people with DM since 1990. The prevalence of DM increased in 131 countries for women and in 155 countries for men. The prevalence of DM is estimated at 828 million (95% CI 757–908) adults for 2040 [1].

On the other hand, the Atlas of the International Diabetes Federation (IDF) for 2023, published in April 2025 [2], estimated that 589 million adults (20–79 years) are living with DM, and this number could increase to 853 million by 2050. Three of four adults with DM are living in low- and middle-income countries. The mortality induced by DM in 2023 was estimated of up to 3.4 million deaths. One trillion USA dollars supposes a 338% increase in healthy expenditure for DM in the last 15 years. One in eight adults (12.5%) is at high risk of developing T2D, and more than 1.8 million children and young adults are living with T1D [2].

These two important studies offer different estimations with regard to the DM prevalence, but, in any case, it seems clear that there is a constant increase of the DM prevalence around the world.

As of 16 August 2025, the number of publications related to DM in PubMed is also constantly increasing, achieving 792,374 publications. The references to cardiovascular (CV) complications of DM were 382,134, 228,533 for Diabetic Kidney Disease (DKD), and 112,065 for diabetic nephropathy (DN) [3].

Although the standardized rates of DM-related complications, acute myocardial infarction, stroke, or amputations, have decreased in the last 15 years, a reduction of the incidence of advanced chronic kidney disease (CKD) requiring renal replacement therapy (RRT) has not been achieved. DKD continues to be the first cause of end-stage renal disease (ESRD) worldwide. Data from the ERA registry of 2022, published in 2025 [4], showed that 23% of the patients with ESKD starting dialysis or requiring a kidney transplant had diabetes as the primary cause of renal disease. The recent data from the REDYT registry, Registro Español de Diálisis y Trasplante, of 2023, found that 25.2% of the patients starting RRT had diabetes as the cause of ESRD [5].

Albuminuria detection, or the estimation of glomerular filtration rate (eGFR), has been the classical method for the diagnosis of CKD, but urinary proteomics analyses are being incorporated for a better early detection of DKD in individuals with baseline eGFR above 60 mL/min/1.73 m^2^.

In addition, various panels of serum and urine biomarkers are in development for an early detection of both diseases [6,7,8].

The clinical picture of the DM patient with advanced CKD has really changed in the last decades, but a very high number of complications are still in progress in these subjects if DM and CKD are not detected in the early stages of both diseases.

Fortunately, multidisciplinary and multifactorial care has effectively improved the ancient catastrophic panorama of the patient with DM and progressive CKD.

The key points of our review are as follows: (1) to remark on the importance of DM as a cause of ESRD; (2) to insist on the integrated multifactorial and multidisciplinary management of DM and CKD for an early diagnosis of both DM and CKD; (3) to show the positive results with the classical drugs for the treatment of these patients; and (4) to show the novel agents that offer renal and vascular protection in the patients with diabetes and/or obesity and/or chronic renal failure.

The timeframe of work of our research extends from 1991 to the present date.

## 2. Integrated Pharmacological Approach for the Management of Patients with DM and CKD

The integrated care of the patients is based on five classes of drugs:

**(2.1) Angiotensin-Converting-Enzyme Inhibitors (ACEI) and Angiotensin II Receptor Blockers (AIIRB)**—demonstrated in several studies with captopril [9], IRMA II [10], IDNT [11] with irbesartan, and RENAAL with losartan [12], their beneficial effect with regard to the reduction of albuminuria and the progression of the renal damage. Thus, renin-angiotensin aldosterone system (**RAAS**) **inhibitors** are maintained as the first-step treatment in the DM patient with hypertension and/or albuminuria or proteinuria (see Table 1).

**(2.2) Sodium-glucose-cotransporter 2** (**SGLT2**) inhibitors have efficiently been added to the usual antidiabetic therapy for adequate metabolic control in subjects with DM but also show a safe CV and nephro-protective profile (see Table 2). The studies of Zinman B et al. [13] and Wanner C et al. [14] with empagliflozin, the CREDENCE trial with canagliflozin [15], the DECLARE and DECLARE-TIMI studies with dapagliflozin [16,17], and many others afterwards have shown the benefits for optimal hypoglycemic management in addition to CV and renal protection.

A meta-analysis by Toyama et al. [18] recruited data from 27 studies with SGLT2 inhibitors. In 7363 patients with T2DM and CKD, SGLT2 inhibitors lowered glycated hemoglobin as well as blood pressure, body weight, and albuminuria. A reduction of the risk of CV death, nonfatal myocardial infarction or nonfatal stroke, and heart failure (HF) was also observed, without a clear effect on all-cause mortality. The study showed an attenuation of the annual decline in the eGFR slope and also a significant risk reduction of the composite renal outcome (HR, 0.71; 95% CI, 0.53–0.95).

Heerspink HJL et al. [19] studied, in a randomized clinical trial (RCT), the effect of dapagliflozin in 4304 patients with CKD with or without DM, with an eGFR of 25 to 75 mL/min/1.73 m^2^ and a urinary albumin-to-creatinine ratio (UACR) of 200 to 5000 mg/g. These patients received dapagliflozin (10 mg once daily) or a placebo. After a median follow-up of 2.4 years, a primary outcome event of a composite of a sustained decline in the eGFR of at least 50%, end-stage kidney disease, or death from renal or cardiovascular causes occurred in 9.2% of patients (*n* = 2152) in the dapagliflozin group and 14.5% of patients (*n* = 2152) in the placebo group (HR, 0.61; relative risk reduction, 39%; *p* < 0.001). The specific renal endpoint was reduced by 44% (*p* < 0.001), and a composite of death from CV causes or hospitalization for HF was reduced by 29% (*p* = 0.009). In addition, 4.7% of patients died in the dapagliflozin group and 6.8% in the placebo group (HR, 0.69; *p* = 0.004), resulting in a 31% relative risk reduction in all-cause mortality. The effects of dapagliflozin were similar in patients with or without DM. New studies are continuously being incorporated on the long-term effects of SGLT2 inhibitors [20,21] (see Table 2).

**Table 2:** Studies with SGLT2 inhibitors.

Name of Study and Authors	Drug	Study Type	N Patients	Patient Type	Results
EMPA-REG, Zinman B et al. [13]	Empagliflozin vs. plac	Ph III RCT	7028 (empa 10 mg, N = 2345, empa 25 mg (n = 2342, plac (n = 2333)))	T2D	Combined 1 EP decreased 12.1% vs. 10.5%, RR 0.86, *p* < 0.001
EMPA REG OTCOME Wanner C et al. [14]	Empagliflozin vs. plac	Ph III RCT	7028 (empa 10 mg, N = 2345, empa 25 mg (n = 2342, plac (n = 2333)))	T2D	Incident nephropathy 12.2% in empag group vs. 18.8% in plac group (HR 0.61, *p* < 0.001). x2 sCreat 1.5% vs. 2.6% (44% RRR). RRT was initiated in 0.3% (empa group) vs. 0.6% (plac group) (RRR 55%). No differences in incident Alb ª.
CREDENCE trial, Mahaffey et al. [15]	Canagliflozin vs. plac	Ph III RCT	4431 T2D	T2D	MACE RR 0.80 (*p* = 0.01), 1 EP (composite of ESRD (dialysis, transplantation, or a sustained estimated GFR of < 15 mL per minute per 1.73 m^2^), a doubling of the s creatinine, or death from renal or cardiovascular causes. RR 0.68 (*p* = 0.01) Second EP RR 0.85 (*p* = 0.25)).
DECLARE, Wiviot SD et al. [16]	Dapagliflozin vs. plac	Ph III RCT	17,160 T2D	T2D	MACE reduction HR 0.93, *p* = 0.17, HF reduction 4.9% vs. 5.8%, hospitalization HR 0.83, *p* = 0.005. Renal event reduction 4.3 vs. 5.6 HR 0.76.
DECLARE—TIMI, Mosenzon O et al. [17]	Dapagliflozin vs. plac.	Ph III RCT	16,863 with albuminuria not CKD	T2D	Reduction of Alb ª and eGFR decline in all categories (*p* < 0.05).
Toyama et al. [18]	SGLT2 inh. vs. plac.	Meta-analysis 27 RCT	7363 T2D + CKD	T2D	Composite 1 EP renal outcomes eGFR decline /dialysis/RTransplant decrease HR 0.71 (29%).
DAPA CKD, Heerspink HJL et al. [19]	Dapagliflozin vs. plac	Ph III RCT	DM + CKD n = 4304. DM = 1455 dapa, 1451 plac.	T2D	Composite 1 EP = or >50% decline eGFR/ESKD/death was 9.2% in dapagl, 14.5% in plac. RR 0.61, *p* < 0.001
Zheng Y et al. [20]	Syst. Rev. and Meta-analysis (20 qualitative and 9 quantitative studies)	RCT	22,313 treated with SGLT2 inh. vs. plac.	T2D with CKD	Strong evidence for protective renal health
Natale P et al. [21]	Syst Rev	53 RCT	65,241 SGLT2 inh. vs. plac.	T2D with CKD	Risk death (2 studies, RRR 0.85–0.94). Renal events: RRR 0.70–0.89 in 2 studies with 12,647 *p*; RRR 0.68–0.78 in 7 studies with 36,380 patients.

SGLT2 I = sodium glucose cotransporter 2 inhibitor. Ph = phase. RCT = randomized clinical trial. T2D = type 2 diabetes. CKD = chronic kidney disease. 1 EP = primary endpoint. 2 EP = secondary endpoint. MACE = major adverse cardiovascular Event. ESRD = end-stage renal disease. RRT = renal replacement therapy. HR = hazard ratio. RRR = relative risk reduction. eGFR = estimated glomerular filtration rate. Alb ª = albuminuria.

**(2.3) Glucagon-like peptide-1 receptor agonists (GLP-1 RAs)** can improve CV and renal events in DKD. These antidiabetic drugs can be safely used without an increased risk of hypoglycemia in various CKD stages in patients with an eGFR above 15 mL/min/1.73 m^2^. These agents have been demonstrated to prevent the onset of albuminuria and to retard the decline of GFR in patients with DM, allowing weight reduction and CV benefits. The effects on body weight sustain these drugs as a solid therapy against obesity [22,23,24]. Currently the GLP-1R agonists are only recommended in type 2 DM.

A variety of RCTs with GLP-1 RA showed positive results in the prevention of new-onset proteinuria [25,26,27,28,29,30] (see Table 3).

Most recently in the FLOW study with 1 mg weekly subcutaneous semaglutide [31], 3533 participants with T2D, with a median follow-up of 3.4 years, were randomized. Low or moderate KDIGO risk was present in 6.8%, while 24.9% had high and 68.3% had very high KDIGO risk. Semaglutide reduced CV death, MI, and stroke by 18% (HR 0.82, *p* = 0.03), with consistency across eGFR categories, UACR levels, and KDIGO risk classification (all *p*-interaction > 0.13). Death due to any cause was reduced by 20% (HR 0.80, *p* = 0.01), with consistency across eGFR and KDIGO risk class (*p* 0.21 and 0.23). The *P*-interaction treatment effect for death due to any cause by UACR was 0.01 [< 300 mg/g HR 1.17; ≥300 mg/g HR 0.70].

**(2.4) The antagonist of the type 1 endothelin receptor** atrasentan has proven to reduce albuminuria and proteinuria. Atrasentan can decrease the risk of kidney failure but at the expense of increasing edema and HF. Proteinuria can be reduced in patients with severe CKD, but the risk of HF may be increased. The effects of atrasentan on kidney and HF events according to baseline eGFR and UACR in a post hoc analysis of the Study of Diabetic Nephropathy with Atrasentan (SONAR) trial have been analyzed by Waijer SW et al. [32]. They studied the effect of atrasentan versus placebo in 3668 patients with T2D and CKD with elevated albuminuria. Atrasentan reduced the RR of the primary kidney outcome, renal composite, and HF hospitalization (HR, 0.71) consistently across all subgroups of baseline eGFR and UACR (*p* > 0.21). Patients in the highest UACR and lowest eGFR subgroups obtained the maximal benefit (*p* < 0.01). The risk of HF hospitalization was higher in the atrasentan group (HR 1.39) (see Table 4).

**(2.5) Finerenone, a non-steroidal mineralocorticoid receptor antagonist (MRA)**, has been tested in RCTs as a kidney protective agent (see Table 5). The phase III studies FIDELIO-DKD [33] and FIGARO-DKD [34], and the pooled analysis FIDELITY [35] in patients with T2D and CKD examined CV and kidney outcomes in different stages of CKD. Among 13,026 patients with a median follow-up of 3.0 years, the composite CV outcome occurred in 12.7% of the patients receiving finerenone and 14.4% in the placebo group (HR, 0.86; *p* = 0.0018). The composite kidney outcome occurred in 5.5% of patients treated with finerenone and 7.1% receiving placebo (HR, 0.77; *p* = 0.0002), a 23% RR reduction. Hyperkalemia as the cause of treatment discontinuation was more frequently observed in patients receiving finerenone (1.7%) than placebo (0.6%).

Ruilope LM et al. [34], in a post hoc analysis of FIGARO-DKD, observed that finerenone reduced the risk of CV events in patients with T2D and stage 3 CKD. FIGARO-DKD included patients with UACR 30 to < 300 mg/g and eGFR 25 to 90 mL/min/1.73 m^2^ or UACR 300 to 5000 mg/g and eGFR ≥ 60 mL/min/1.73 m^2^. A decrease of > 40% in the eGFR was observed at a lower rate with finerenone compared with placebo (HR = 0.87; *p* = 0.069). The treatment with finerenone was associated with an RR of 23% (HR 0.77 (*p* = 0.041)), if an eGFR reduction of > 57% was considered. These RCTs did not include any parameter or biomarker to evaluate the effect on fibrosis. In patients with severely increased albuminuria, a more intense effect of finerenone was observed. Improvements in UACR, eGFR slope, and CV risk were evident in both subgroups with finerenone.

In a very recent study, Agarwal R et al. [36] have analyzed the effect of finerenone (10 or 20 mg/day) (N = 264), empagliflozin (10 mg/day, N = 267), or the combination of the two drugs in an RCT in patients with eGFR 30 to 90 mL/min/1.73 m^2^, UACR of 100 to = or <5000 mg/g, and T2D. The initial combination with finerenone-empagliflozin led to a greater reduction in the UACR than either treatment alone.

Patients with DM and CKD are at very high risk of developing kidney failure, atherosclerotic CV disease, HF, and premature death.

The Kidney Disease Improving Global Outcomes (KDIGO) 2022 [37] and the 2022 American Diabetes Association (ADA) Standards of Medical Care in Diabetes and the Clinical Practice Guideline for Diabetes Management in Chronic Kidney Disease [38] have added evidence-based recommendations. The new ADA 2025 Guidelines for the standard care of DM [39] insist on some aspects that should be taken into consideration when comparing to the Guidelines of 2024: (1) Diagnostics may consider antibody-based screening for presymptomatic T1D in individuals with a family history of the disease. (2) GLP-1 RAs or dual GIP-GLP-1 RAs are recommended for their multifaceted benefits in diabetes management. (3) Adequate water consumption, high-quality and sustainable eating patterns such as plant-based diets, and a recommendation to replace sugar with non-nutritive sweeteners in moderation and short-term to facilitate caloric restriction may be offered as dietary guidance. (4) Digital technology powered by the systems or a continuous glucose monitoring (CGM) for individuals with T2D on non-insulin regimens as well as those on insulin may be implemented.

## 3. New Emerging Molecules for the Management of Patients with CKD and/or DM and/or Obesity (See Table 6, Table 7, Table 8, Table 9 and Table 10)

While our thoughts were yet installed into the future for the management of patients with DM and DKD [40], the future is being constantly surpassed. Many new drugs of different therapeutic groups are now in development and may be added to the five classical pillars described before, but also for the treatment of patients with CKD without DM. Numerous RCTs are currently in progress and will offer surprising results in the next months and years. These novel drugs include other non-steroidal mineralocorticoid receptor antagonists, such as balcinrenone [41]; aldosterone synthase inhibitors, such as baxdrostat [42] and vicadrostat [43]; different GLP-1 RAs, such as tirzepatide [44], survodutide [45], and retatrutide [46]; new endothelin receptor antagonists, such as zibotentan [47]; and soluble guanylate cyclase activators, such as avenciguat [48].

Many patients with HF have CKD and may not tolerate MRA. Lam CSP et al. investigated the efficacy and safety of the mineralocorticoid receptor modulator balcinrenone in combination with dapagliflozin in a phase II study [41].

From January 2021 to October 2023, 133 adults with symptomatic HF, ejection fraction < 60%, eGFR ≥ 30 to ≤ 60 mL/min/1.73 m^2^, and UACR ≥ 30 to < 3000 mg/g were randomized to receive balcinrenone 15, 50, or 150 mg/day plus dapagliflozin 10 mg/day, or dapagliflozin 10 mg/day plus placebo, for 12 weeks. Enrollment was stopped early because of slow recruitment. Relative reductions in UACR from baseline to week 12 (primary endpoint) were not significantly different between the balcinrenone plus dapagliflozin groups versus dapagliflozin plus placebo. There was no clear balcinrenone dose–response relationship. Possible dose-dependent increases in serum potassium levels, reduced eGFR in the highest dose group, and non-significant trends towards reduced N-terminal pro-B-type natriuretic peptide levels were observed. Hyperkalemia led to discontinuation in two participants treated with balcinrenone plus dapagliflozin and none in those who received dapagliflozin plus placebo. The study did not show a significant reduction in UACR, probably due to the small sample size [41].

**Table 6:** Summary of a novel study with an emerging mineralocorticoid receptor antagonist, balcinrenone, for the treatment of patients with chronic kidney disease and heart failure.

Name of Study and Authors	Drug	Study Type	N Patients	Patient Type	Results
MIRACLE, Lam CSP et al. [41]	Balcinrenone (10.50 or 150 mg/d) + Dapagliflozin 10 mg/d Vs. Dapag 10 g/d + plac.	Ph II RCT	166 planned, not achieved	CKD + symptomatic HF	Stopped early because of low recruitment. No dose-dependent influence sK was observed No UACR decrease at 12 weeks.

CKD = chronic kidney disease. HF = heart failure. Ph = phase. RCT = randomized clinical trial. sK = serum potassium. UACR = urine albumin to creatinine ratio.

Baxdrostat is a selective small-molecule aldosterone synthase inhibitor in development to treat hypertension and CKD. In a phase I, open-label, parallel-group study [42], the safety and pharmacokinetics (PK) of baxdrostat were assessed in participants with varying degrees of renal function. Three groups of individuals were included in the study: controls (eGFR ≥ 60 mL/min/1.73 m^2^), patients with moderate to severe renal impairment (eGFR 15–59 mL/min/1.73 m^2^), or those with stage 5 CKD (eGFR < 15 mL/min/1.73 m^2^) and received a single 10-mg baxdrostat dose. Pharmacokinetic blood and urine samples were analyzed at 7 days. Thirty-two participants completed the study. No patient died, and only one mild diarrhea adverse event was registered. No clinically significant changes in laboratory parameters, vital signs, physical examinations, or ECGs were observed. Plasma concentration–time curves of baxdrostat were similar among all groups. Urine PK parameters (12% excreted) in the moderate to severe renal impairment and control groups were similar. Minimal urinary baxdrostat excretion was reported in the renal failure group. Renal impairment had no significant impact on systemic exposure or clearance of baxdrostat. This fact suggests that dose adjustment due to PK differences in patients with kidney disease is probably not necessary.

A phase II study in 586 patients with albuminuric CKD has shown that 10 mg of vicadrostat (BI690517) [43], another aldosterone synthase inhibitor, reduced UACR by 40% compared with placebo, with or without associated empagliflozin treatment. Its use added to an SGLT2i may decrease the risk of hyperkalemia, improving tolerability and allowing the treatment of more patients, including those with higher levels of blood potassium. This approach will be tested in the EASi-KIDNEY (NCT06531824), a phase III double-blind placebo-controlled trial, by assessing the safety and cardiorenal efficacy of vicadrostat in combination with empagliflozin in 11,000 patients with CKD and with or without diabetes.

**Table 7:** Aldosterone-synthase inhibitors for the treatment of chronic kidney disease.

Aldosterone-Synthase Inhibitors					
Name of Study and Authors	Drug	Type of Study	Number of Patients	Type of Patients	Results
Freeman MW et al. [42]	Baldrostat 10 mg/d by 7 days	Ph I pharmacokinetic study	N = 32	CDK diverse stages	Renal impairment had no significant impact on systemic exposure or clearance of baxdrostat. Dose adjustment due to PK differences in patients with kidney disease is probably not necessary
EASi KIDNEY, Judge PK et al. [43]	Vicadrostat 10 mg/d (+RAAS inh + empagliflozin 10 mg/d) vs. plac	Ph III RCT Runnning st.	Stratum 1: 4800 p. Stratum 2; 6200 p. Follow-up 3 y.	CKD	1070 outcomes are expected in 3 years in each group. 1 EP: composite kidney progression and 2 EP: composite CV death/HF hospitalization

RAAS = renin-angiotensin II-aldosterone system. Ph = phase. RCT = randomized clinical trial. CKD = chronic kidney disease. 1 EP = primary endpoint. 2 EP = secondary endpoint. CV = cardio vascular. HF = heart failure.

CVD and DKD biomarkers with off-label long-term (21 months) use of tirzepatide in overweight (OW) or obese (OB) adults with T1D were evaluated in a retrospective chart review study [44], and data from 84 OW/OB adults with T1D who received tirzepatide since July 2022 and were treated for a minimum of 6 months were analyzed. A control group (*n* = 38) was matched for age, DM duration, sex, glycosylated hemoglobin (HbA1c), and body mass index (BMI). Data were collected electronically over 21 months of treatment. eGFR over time linear mixed effects models were used to examine the changes in lipids and blood pressure. Tirzepatide users had a slightly higher baseline BMI than controls, 35.2 ± 4.8 kg/m^2^ and 33.3 ± 4.2 kg/m^2^ (*p* = 0.03), respectively. Weight loss (−59 ± 4.6 lbs. [−23.4%]) was higher in the tirzepatide-treated group compared with a gain (+1.7 ± 5.0 lbs. [+1.8%]) in controls. The HbA1c decreased more in patients using tirzepatide than in controls (−0.50 ± 0.07% and −0.24 ± 0.09%, respectively, *p* = 0.017). Tirzepatide-treated patients significantly improved triglycerides, total and low-density lipoprotein cholesterol, systolic blood pressure, and eGFR, changes that remained significant after adjusting for weight and HbA1c. The eGFR declined significantly in controls but not in the tirzepatide group. The authors concluded that long-term use of tirzepatide in OW/OB adults with T1DM results in more than 23% weight loss and sustained improvement in glucose control. A significant improvement in cardiovascular biomarkers and preservation of kidney function was observed, independently of changes in weight and/or HbA1c. Nevertheless, we must say that currently tirzepatide has no indication in T1D.

The efficacy and safety of survodutide, a GLP-1 dual receptor agonist, in people with a BMI ≥ 27 kg/m^2^ was tested in 387 individuals (aged 18–75 years, BMI ≥ 27 kg/m^2^, without DM and with no CKD) who were randomized 1:1:1:1:1 to once-weekly subcutaneous survodutide (0.6, 2.4, 3.6, or 4.8 mg) or placebo for 46 weeks (20-week dose escalation; 26-week dose maintenance). Patients were categorized according to sex and baseline BMI. Data were analyzed descriptively in accordance with the dose assigned at randomization using on-treatment data or all data censored for COVID-19-related treatment discontinuation (ClinicalTrials.gov number: NCT04667377). After 46 weeks of treatment, females had greater reductions in body weight and waist circumference than males. Participants with a lower baseline BMI had greater proportional reductions in body weight than those with a higher baseline BMI. The trend was reversed for reductions in waist circumference. Rates of adverse events were comparable between subgroups for sex and baseline BMI. The most frequently reported gastrointestinal effect was nausea in all subgroups [45].

**Table 8:** Studies with glucagon-like-peptide 1 receptor agonists for the treatment of diabetes, obesity, or chronic kidney disease.

GLP-1 R Agonists					
Name of Study and Authors	Drug	Type of Study	Number of Patients	Type of Patients	Results
Garg SK et al. [44]	Tirzepatide vs. controls	Retrospective Ph IV	T1D n = 84 Controls n = 38	T1D + BMI = or >27 kbw/1.73 m^2^	Reta had greater weight loss, better renal function, fewer proinflammatory cytokines in the kidneys, and better lipid profiles, as compared to Tirze and Lira. Tirze had better blood glucose lowering, weight loss, and lipids as compared to Lira
Le Roux et al. [45]	Survodutide Sc 0.6/2.4/3.6/4.8 mg/w, dose escalating vs. plac	Ph II	N = 387	Overweight or obesity	In people with a BMI ≥ 27 kg/m^2^, survodutide significantly reduced body weight and waist circumference when compared with placebo in prespecified subgroups based on sex and baseline BMI and was tolerated at all doses tested.
Ma et al. [46]	10 nmol/kbw intraperitoneal injection of Liraglutide vs. Tirzepatide vs. Retatrutide	Experimental T2D model	10 Db/db mouse per group	Experimental db/db mouse	Reta was superior in reducing weight, improving renal function in db/db mice, and suppressed the expression of pro-inflammatory cytokines and pro-fibrotic factors in the kidneys of mice. Reta enhanced liver function, improved all lipidic parameters, and increased intestinal metabolite butyrate in db/db mice. Tirze exhibited better effects on lowering blood glucose, weight loss, lipid reduction, and improvement of DKD compared to liraglutide.

T1D = type 1 diabetes. T2D = type 2 diabetes. Db/db = diabetic/nondiabetic alleles. BMI = body mass index. Kbw = kg body weight. Lira = liraglutide. Reta = retatrutide. Tirze = tirzepatide.

With regard to a new GLP-1 RA, a study was conducted in db/db mice, an experimental model of T2D, to assess and compare the therapeutic efficacy of liraglutide, tirzepatide, and retatrutide in treating DKD [46]. Experimental animals were administered intraperitoneal injections of liraglutide (10 nmol/kg), tirzepatide (10 nmol/kg), and retatrutide (10 nmol/kg) for 10 weeks. The effectiveness of these three drugs in controlling blood glucose levels, reducing weight, and improving serum biochemical indicators and DKD was tested. Renal inflammation and fibrosis indexes were measured and compared. The content of intestinal metabolite butyrate was compared to reflect the regulatory effects of these three drugs on gut microbiota.

Retatrutide demonstrated superior effectiveness in reducing weight and improving renal function in db/db mice compared to liraglutide and tirzepatide. The expression of pro-inflammatory cytokines (TNF-α, caspase-1, and NLRP3) and pro-fibrotic factors (fibronectin, α-SMA, and collagen I) was significantly suppressed in the kidneys of mice. Retatrutide substantially enhanced liver function, reduced triglyceride and cholesterol levels, low-density lipoprotein cholesterol, elevated high-density lipoprotein cholesterol, and increased the content of intestinal metabolite butyrate when compared to the other two drugs. But despite its ability to lower blood glucose levels, retatrutide was not superior for blood glucose control. Tirzepatide showed better effects on lowering blood glucose, weight loss, lipid reduction, and improvement of DKD compared to liraglutide. The authors concluded that retatrutide and tirzepatide were significantly effective in improving DKD and controlling blood glucose and body weight. Retatrutide was the most effective in improving DKD and body weight, while tirzepatide was the most effective in controlling blood glucose. The reason we included the results of an animal experimental study in our review comparing the effects of these drugs lies in the benefits of these agents on renal and liver function, which could probably be applied to the human beings in subsequent RCTs.

In the same way, we wish to include some studies with regard to obesity, due to its frequent association with T2D and CKD and its impact on cardiovascular and kidney complications.

Heerspink HLJ et al. [47] conducted a double-blind, active-controlled, Phase IIb study to evaluate the efficacy and safety of the endothelin A receptor antagonist zibotentan in combination with the SGLT2 inhibitor dapagliflozin in 415 adults with CKD (Zibotentan and Dapagliflozin for the Treatment of CKD; ZENITH-CKD). Participants are being randomized (1:2:2) to zibotentan 0.25 mg plus dapagliflozin 10 mg once daily, zibotentan 1.5 mg plus dapagliflozin 10 mg and dapagliflozin 10 mg alone for 12 weeks, followed by a 2-week off-treatment washout period. The primary endpoint is the change in log-transformed UACR from baseline to week 12. Secondary objectives include change in blood pressure from baseline to week 12 and change in eGFR. Other defined events will include changes in weight gain or B-type natriuretic peptide levels. At baseline 447 patients were randomized and received treatment in placebo/dapagliflozin (*n* = 177), zibotentan 0.25 mg/dapagliflozin (*n* = 91), and zibotentan 1.5 mg/dapagliflozin (*n* = 179). The mean age was 62.8 years; 30.9% were female, and 68.2% were white. The mean baseline eGFR of patients was 46.7 mL/min/1.73 m^2^, and the geometric mean UACR was 538.3 mg/g. This study will evaluate the UACR-lowering efficacy and safety of zibotentan with dapagliflozin as a new treatment for CKD.

At 12 weeks, UACR decreased by −33.7% (*p* < 0.001, in the zibotentan 1.5 mg group) and −27% (*p* = 0.0022) in the zibotentan 0.25 group, all groups vs. dapagliflozin plus placebo. The percentage of fluid retention was 18% in zibotentan 1.5-mg-treated patients, 9% in zibotentan 0.25 mg, and 8% in dapagliflozin plus placebo. This study is continuing in a phase III trial to test the dose efficacy and safety for a long-term follow-up.

**Table 9:** A novel endothelin 1 receptor antagonist for the treatment of chronic kidney disease.

Endothelin A Receptor Antagonists					
Name of Study and Authors	Drug	Type of Study	Number of Patients	Type of Patient	Results
ZENITH-CKD Heerspink HJL et al. [47]	Zibotentan 1.5 mg/d + Dapagliflozin 10 mg/d; Zibot 0.25 mg/d + dapaglif 10 mg/d; Dapagliflozin 10 mg/d + plac.	Ph IIb RCT	CKD (n = 449 zibot 1.5 + dapa); (n = 91 zibot 0.25 + dapa) N = 177 dapa + plac	CKD	At 12 w: UACR decrease −33.7% (*p* < 0.001, in zibotentan 1.5 mg group); −27% (*p* = 0.0022) in zibot. 0.25 group, all groups vs. dapa + plac. Fluid retention: 18% in zibot. 1.5 mg; 9% in zibot 0.25 mg; 8% in dapa + plac.

CKD = chronic kidney disease. Ph = phase. RCT = randomized clinical trial. UACR = urine albumin to creatinine ratio.

Avenciguat is a novel, potent soluble guanylate cyclase activator in development for CKD. Two trials investigated avenciguat in diabetic (NCT04750577) and non-diabetic (NCT04736628) CKD. A prespecified pooled analysis of two randomized, double-blind, placebo-controlled trials [48] has included adults with CKD (eGFR ≥ 20 and < 90 mL/min/1.73 m^2^ and UACR ≥ 200 and < 3500 mg/g). The patients were randomized to 20 weeks of placebo or avenciguat 1, 2, or 3 mg three times daily (TID), associated with ACEI or AIIRB. The primary endpoint was change from baseline in UACR in 10 h urine at week 20. The secondary endpoint was UACR change from baseline in first morning void urine (FMVU) at week 20. Five hundred patients (mean age 62 +/− 13 years; mean eGFR 44 +/− 18 mL/min/1.73 m2 and median 10 h UACR 719 [379–1285] mg/g) were treated with avenciguat 1 mg (n = 125), 2 mg (n = 126), or 3 mg (n = 127), or placebo (n = 122). DM affected all 243 patients in the first study and 27 of 261 patients in the second. Avenciguat 1, 2, and 3 mg reduced UACR in 10-h and FMVU versus placebo throughout the treatment period. At week 20, placebo-corrected geometric mean changes (95% C.I.) from baseline in UACR in 10 h urine with avenciguat 1, 2, and 3 mg were −15.5% (−26.4 to −3.0), −13.2% (−24.6 to −0.1), and −21.5% (−31.7 to −9.8), respectively. Corresponding changes in FMVU were −19.4% (−30.0 to −7.3), −15.5% (−26.9 to −2.5), and −23.4% (−33.5 to −11.8), respectively. The overall frequency of adverse events was low and similar to placebo. The number of patients who discontinued the study drug due to adverse events with avenciguat 1, 2, and 3 mg TID were five (4%), 11 (9%), and 11 (9%), respectively, compared with four (3%) in the placebo group.

**Table 10:** The guanylate-cyclase activator avenciguat for the treatment of chronic kidney disease.

Guanylate Cyclase Activators					
Name of Study and Authors	Drug	Type of Study	Number of Patients	Type of Patient	Results
Heerspink JHL et al. [48]	Avenciguat 1, 2 or 3 mg/TID vs. plac.	Ph III RCT, studies 1 and 2 Pooled analysis	500 CKD patients, (DM = 243 in study 1 and 27 in study 2).	CKD or CKD + T2D.	UACR in 10 h urine: −15.5% (avenciguat 1 mg), −13.2% (avenciguat 2 mg) and −21.5% (avenciguat 3 mg)- UACR in first morning void urine: −19.4% (avenciguat 1 mg), −15.5% (avenciguat 2 mg) and −21.4% (avenciguat 3 mg)

DM = diabetes mellitus. CKD = chronic kidney disease. Ph = phase. RCT = randomized clinical trial. UACR = urine albumin to creatinine ratio. T2D = type 2 diabetes. TID = three times per day.

New potential horizons for the treatment of DKD and also of CKD without DM, such as strategies based on actions on gut microbiota or stem cell therapies, are expected in the next months or years.

## 4. Discussion

Chronic kidney disease coexisting with diabetes, in the absence of other clear causes of kidney injury, occurs in approximately 20 to 40% of patients with diabetes mellitus [49]. The pathophysiology of the renal damage produced in the context of diabetes is very complex, and many different mechanisms and factors are involved.

In this current review we have tried to summarize the pharmacological management of the patients with diabetes associated with chronic kidney disease in the last three decades—from 1990 up to date—independent of the presence of obesity, which is very frequently associated with both DM and CKD. The catastrophic panorama of the patients with diabetes and CKD, who achieved the nephrology care years ago, has really improved in the last decades due, on one hand, to a more integrated multifactorial and multidisciplinary care that has proven to be effective for the early detection of both DM and CKD. But, on the other hand, different classes of combined drugs are used for the control of glycemia and hypertension that may have protective effects on CV risk factors and contribute to slowing the progression of CKD [9,10,11,12,13,14,15,16,17,18,19,20,21,22,23,24,25,26,27,28,29,30,31,32,33,34,35,36]. The classical pillars of pharmacological care have been described on the basis of many of the randomized clinical trials that have generated evidence for metabolic control and cardiovascular and renal protection. A summary of the principal results of these studies has been expressed in Table 1, Table 2, Table 3, Table 4, Table 5, Table 6, Table 7, Table 8, Table 9 and Table 10.

In addition to the application of the standards of care and the clinical practice guidelines for the patients’ management [6,38,39,40], the combination of some new molecules in adequate personalized doses offers a better way for an integrated management of patients with DM and CKD. Many novel therapeutic agents to treat diabetes, obesity, heart failure, or chronic kidney disease are in development. The approach that includes different specialists, patients, and health providers working together in multidisciplinary teams and supplying educational programs may lead to an early diagnosis of both DM and CKD. An optimal time to refer the patient with DM and renal involvement to nephrology care should be the key point to coordinate the integrated multifactorial management of our patients [6,50]. The principal strength of this article relies on the analysis of positive results with the drugs that have generated clinical evidence from 1991 and the most recent publications developed in randomized clinical trials with the novel agents used for the control of glycemia and/or obesity in the context of chronic kidney disease. Our review has clear limitations. It is an overview of the most recent advances in this context, but, taking into account the increasing number of articles continuously published in this regard, this review can be immediately surpassed by new evidence, because there are important ongoing RCTs whose outcomes will be presented in a short or medium time.

## 5. Conclusions

Diabetes mellitus continues to be a global world health problem. It is the principal cause of ESRD. An optimal time to refer the patient with DM and renal involvement to nephrology care should be the key point to coordinate the integrated multifactorial management of our patients. If we are able to apply these combined measures, including personalized doses of classical and novel molecules, perhaps in a next step we should be able to “fold the curve” and to slow the progression to ESRD and the CV damage in the patients with DM, allowing us to definitively decrease DM as the first cause of advanced CKD.

## Figures and Tables

**Table 1:** Summary of studies with RAAS inhibitors.

Name of Study and Authors	Drug	Study Type	N Patients	Patient Type	Results
***RAAS Blockers***					
Captopril trial, Lewis EJ et al. [9]	Captopril vs. placebo	Ph III RCT	409 (207 capt, 202 plac)	T1D	50% decrease combined 1 EP (x2 sCreat, dialysis, renal transpl)
IRMA II, Parving HH et al. [11]	Irbesartan vs. plac	Ph III RCT	590	T2D	1 EP (doubling of the base-line serum creatinine concentration) (HR 0.30, *p* < 0.001)
IDNT, Lewis EJ et al. [10]	Irbesartan vs. amlodipin vs. plac	Ph III RCT	1715	T2D	x2 sCreat decrease 21% vs. 24% (vs. plac *p* < 0.03, vs. amlodi *p* < 0.05)
RENAAL, Brenner BM et al. [12]	Losartan vs. plac	Ph III RCT	1513	T2D	x2 sCreat decrease 16% (*p* = 0.006), decrease ESRD 28%/(*p* = 0.02), decrease prot ª 35% (*p* = 0.001)

RAAS = renal-angiotensin II-aldosterone system. Ph = phase. RCT = randomized clinical trial. T1D = type 1 diabetes. T2D = type 2 diabetes. 1 EP = primary endpoint. HR = hazard ratio. ESRD = end-stage renal disease. prot ª is a shorten expression of proteinuria.

**Table 3:** Some studies with GLP-1 R agonists.

Name of Study and Authors	Drug	Study Type	N Patients	Patient Type	Results
STEP 1 to 8 propramme. Bergman NC et al. [22]	Review	8 RCT	Semaglutide 2.4 mg sc/week N = 1961 pat.	Obesity without DM	At week 68, 17.4% (semagl. group) vs. 14.9% (plac) weight loss.
SURMOUNT-1, Jastreboff et al. [23]		2539 p	2539 (1032 with Pre DM)	Obesity	At week 17, 1.3% in tirzepatide group vs. 13.3% in plac group developed T2D, HR 0.12, *p* < 0.001
Davies MJ et al. [24]	Cagrilintide—semaglutide vs. plac	1206	Cagrilintide +semag (n = 904), placebo (n = 342)	Obesity+T2D	At week 68, −13.7% (cagri + semagl) vs. −3.4% (plac) weight loss.
ELIXA Musquiet ME et al. [25]	Lixisenatide vs. plac	6068 5978 microAlb ª available	Lixienatide 2250 lixisenatide vs. 2191 plac.	T2D	At week 108, % change in Alb ª: −1.69% in normoAlb ª (*p* = 0.73); −21.1% in microAlb ª (p0.05) and −39.18% in macroprot ª (*p* = 0.007)
EXSCEL Holman HH et al. [26]	Exenatide 2 mg/sc/w vs. plac		N = 14,752 (10,782 with previous CV disease)	T2D	1 composite EP 11.4% in the exenatide group and 12.2% in the placebo (HR 0.91). Exenatide safety was noninferior (*p* < 0.001) but was not superior to efficacy (*p* = 0.06 for superiority). Death from CV causes, hospitalization for heart failure or acute coronary syndrome and serious adverse events did not differ significantly
LIRA RENAL Davies MJ et al. [27]	Lixisenatide vs. plac	Ph III RCT	Lixisenatide sc n = 279	T2D + CKD	Weight loss −2.41 kbw vs. −1.09 (*p* < 0.0052). No changes in renal function.
LEADER, Marso SP et al. [28]	Liraglutide vs. plac	Ph III RCT	N = 94,340, Liraglutide (4668) vs. plac (n = 4672)	T2D	Composite 1 EP 13% vs. 14.9%, RR 0.87, *p* < 0.001. Death from CV causes 4.7 vs. 6% (RR 0.78, *p* < 0.007); deaths from any cause 8.2 vs. 9.6%, RR 0.85, *p* = 0.02
REWIND Gerstain HC et al. [29]		Ph III RCT	Dulaglutide 1.5 mg sc/w. Dukagl n) 4949 vs. plac (n = 4952)	T2D with previous CV risk factors	Composite 1 EP 12% vs. 13.4% HR 0.88, *p* = 0.026. No differences on mortality rate.
AWARD Tuttle K et al. [30]	Dulaglutide vs. insulin glargine	Ph III RCT	Dulaglutide n = 577, 1.5 mg sc/w, dulag 0.75 mg sc/w vs. insulin glargine	T2D + CKD	At 52 w: eGFR dulaglutide groups: 34 mL/min/1.73 m^2^; eGFR in insulin glargine 31 mL/min/1.73 m^2^ (< 0.005). UACR and glucose control without differences between groups.
FLOW Mahaffy et al. [31]	Semaglutide 1 mg sc/week 52 w	Ph III RCT	N = 3533 KDIGO low risk (n = 242 p), high risk (n = 878 p), very high risk (n = 2412)	T2D + CKD	Decrease in CV death/myoc infart c/stroke 18% in semaglutide patients, regardless of baseline CKD severity.

Ph = phase. RCT = randomized clinical trial. T2D = type 2 diabetes. CKD = chronic kidney disease. 1 EP = primary endpoint. 2 EP = secondary endpoint. ESRD = end-stage renal disease. HR = hazard ratio. RR = relative risk. UACR = urine albumin to creatinine ratio. eGFR = estimated glomerular filtration rate. Alb ª = Albuminuria.

**Table 4:** Studies with endothelin A receptor antagonists.

Name of Study and Authors	Drug	Study Type	N Patients	Patient Type	Results
SONAR Waijer et al. [32]	Atrasentan 1 mg/d vs. plac	Ph III RCT	Initial phase n = 5117; enrichment phase n = 3668 p	T2D + CKD	1 EP decrease HR 0.71, highest benefit in patients with higher UACR and lower eGFR. High risk of hospitalization for HF across all categories of UACR and eGFR.at baseline

Ph = phase. RCT = randomized clinical trial. T2D = type 2 diabetes. CKD = chronic kidney disease. UACR = albumin urine to creatinine ratio. eGFR = estimated glomerular filtration rate.

**Table 5:** Studies with mineralocorticoid receptor antagonists.

	Drug	Study Type	Number of Patients	Type of Patient	Results
FIDELIO DKD, Bakris G et al. [33]	Finerenone vs. plac	Ph III RCT	N = 5734, 2833 finerenone, 2841 plac.	T2D + CKD	Combined 1 EP 17.8% vs. 21.1% HR 0.86, *p* = 0.001; 2 EP kidney failure/sustained decrease eGFR/death renal cause 13% vs. 14.8% HR 0.86, *p* = 0.03
FIGARO Ruilope JM et al. [34]	Finernone vs. plac	RCT	7352	T2D + CKD	Higher effects on the eGFR decrease < 57% composite 1 EP HR 0.77, *p* = 0.041, RRR 36% for ESRD
FIDELITY Agarwal R et al. [35]	Finerenone vs. plac	Pool analysis 2 RCT	N = 6519 finerenone N = 6507 plac	T2D + CKD	Comp 1 EP CV outcome 12.7% vs. 14.4%, HR 0.86, *p* = 0.00018. Composite kidney outcomes: 5.5% vs. 7.1%, HR 0.77, *p* = 0.0002
Agarwal R et al. [36]	Finerenone 10 mg/d, empagliflozin 10 mg/d, or combined finerenone + empagliflozin	Ph III RCT	Finerenone n = 258 *p*; empagliflozin n = 261 *p*, combined t n = 265 *p*	T2D + CKD	At day 180, there was a reduction in the UATC ratio with combination therapy (HR 0.71; *p* < 0.001) and also greater than with empagliflozin alone (0.68; *p* < 0.001)

Ph = phase. RCT = randomized clinical trial. T2D = type 2 diabetes. CKD = chronic kidney disease. 1 EP = primary endpoint. 2 EP = secondary endpoint. ESRD = end-stage renal disease. HR = hazard ratio. RRR = relative risk reduction. eGFR = estimated glomerular filtration rate.

## Data Availability

The original contributions presented in this study are included in the article. Further inquiries can be directed to the corresponding author.
